# New targets for the treatment of ulcerative colitis: Gut microbiota and its metabolites

**DOI:** 10.1016/j.csbj.2025.05.006

**Published:** 2025-05-09

**Authors:** Huanyu Li, Meng Pan, Yifan Li, Manli Cui, Mingxin Zhang

**Affiliations:** a Second Clinical Medical College, Shaanxi University of Chinese Medicine, Xianyang, Shaanxi 712046, China; b College of Basic Medical Sciences, Shaanxi University of Chinese Medicine, Xianyang, Shaanxi 712046, China; c Department of Gastroenterology, the First Affiliated Hospital of Xi’an Medical University, Xi’an, Shaanxi 710077, China

**Keywords:** Gut microbiota, Microbial metabolites, Ulcerative colitis, Microcapsules, Nanoparticles, Prebiotics, Probiotics, FMT

## Abstract

Ulcerative colitis (UC) is one of the most common and difficult-to-treat inflammatory diseases Currently, the standard of care includes immunological modulation and anti-inflammatory medication to alleviate symptoms; however, these treatments are associated with several side effects. As a result, developing novel, safe, and effective treatment strategies is crucial. The gut microbiota and its influence on the onset and progression of UC through their regulation of immunity, barrier integrity, and homeostasis, serves as a promising target for UC therapy. In this review, we explore the pathological changes that take place in UC along with the role of gut microbiota and its metabolites in disease progression and modulation. Additionally, we offer a thorough description of novel UC treatment approaches that focus on altering the gut microbiota and its metabolites. These protocols include FMT, probiotics, prebiotics, and micro/nanoparticles. The ultimate goal is to offer a theoretical basis for the advancement of innovative therapeutic strategies for UC.

## Graphical Abstract

**Figure:**
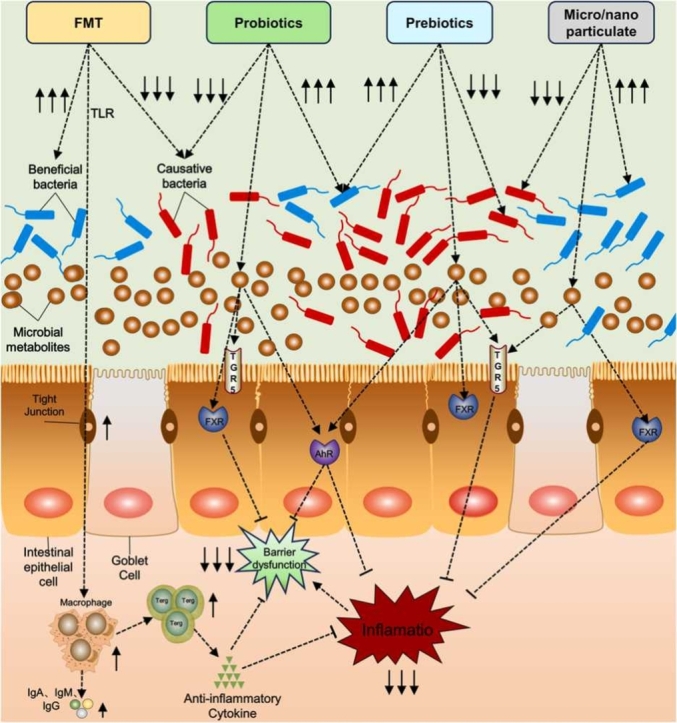

## 1 Introduction

UC is characterized by congestion, erosion, and ulceration of the colorectal mucosa. Clinically, it presents with recurrent abdominal pain, diarrhea, and mucopurulent and bloody stools, negatively impacting patients' quality of life[1]. Over the past two decades, the incidence of UC has been on the rise, with recent surveys indicating an incidence rate of 9–20 cases per 100,000, and a total prevalence of 156–291 cases per 100,000[2]. In the United States, there are around two million annual UC cases, with medical costs estimated to exceed $4 billion each year, imposing a heavy health economic burden on society[3]. Current treatment options for UC include aminosalicylates, glucocorticoids, immunomodulators, and biologics, which aim to suppress the immune response and inflammatory response[4], [5]. However, the adverse effects and high recurrence rates associated with the long-term use of these medications have limited their effectiveness[6], [7]. This presents a compelling need for more comprehensive studies on the initiation and progression of UC to explore new directions and methods. Research highlighted a significant connection between the pathophysiology of UC and the dysbiosis of the gut microbiota along with associated metabolites [8], [9], [10]. Compared to healthy individuals, the gut microbiota of patients with UC exhibits notable alterations. Studies have shown that transplanting UC-associated dysregulated gut microbiota into healthy mice can disrupt their gut bacterial composition and exacerbate colitis[11]. Conversely, antibiotic interventions have been found to alleviate colitis symptoms in susceptible individuals[12]. These outcomes can be attributed to evidence that changes in gut microbiota composition correspond to changes in their metabolites. These small molecule metabolites play a crucial role in signaling processes within the intestine, which is essential for maintaining microenvironmental homeostasis[13]. The gut microbiota and its metabolites can modulate the immune system in various ways, such as promoting the growth of immune cells and facilitating appropriate immune responses [14]. Additionally, the normal intestinal microbiota and its metabolites work as a microbial barrier to prevent the entry of foreign bacteria and limit the spread of harmful microbes[15]. Furthermore, the microbiota in the intestines and its metabolites have an enormous effect on regulating human nutrition and metabolism [16], [17]. When factors such as environment, diet, and medications cause dysbiosis, it can lead to pathogenic bacteria colonizing the intestines, and triggering colonic inflammation through immune modulation, driving the development of UC. Dysbiosis can disrupt the release of metabolites, seriously affecting intestinal immunity and the gut barrier's functionality. Consequently, it is becoming commonplace to treat UC by modifying the gut microbiota and its metabolites. The article discusses the benefits of having a healthy gut microbiota and its metabolites. We will outline how the gut microbiota and its metabolites change in cases of UC along with the resulting consequences. To pave the way for the creation of new clinical protocols for treating UC, we will conclude by summarizing the key studies conducted in the past ten years on treating UC through the modification of gut microbiota and its metabolites.

## 2 Gut microbiota protects the intestines

The gut microbiota consists of approximately 100 trillion bacteria, viruses, fungi, protists, and archaea. Among these, more than 90 percent are Bacteroides and Firmicutes, with Actinobacteria, Proteobacteria, and Clostridium close behind. These microbes are responsible for various physiological processes, including digestion, absorption, metabolism, and host immunity, and they create a microbial barrier in the gut[18] [19]. One of the primary functions of gut microbiota is to nourish the intestinal lining cells. They aid in the digestion of certain nutrients and help decompose some indigestible substances, such as dietary fiber and protein, converting them into elements that can be absorbed by the intestine, thus ensuring the nutrition and energy supply for intestinal cells[20]. Additionally, gut microbiota produces essential metabolites such as bile acids (BAs), tryptophan (Trp), and short-chain fatty acids (SCFAs). Once absorbed by the intestine, these metabolites act as signaling molecules and substrates of metabolic reactions, which are vital for maintaining normal physiological function in the host’s intestines[21].

### 2.1 Gut microbiota are necessary for building the intestinal mucosal immune system

Research has shown that the gut immune system of germ-free (GF) animals is immature compared to that of specific pathogen-free (SPF) animals[22]. This immaturity is evidenced in GF mice, which was shown by fewer and smaller mesenteric lymph nodes, isolated lymphoid follicles, and Peyer's patches. However, introducing specific gut flora has been shown to improve these conditions[23]. This indicates that gut microbiota plays a crucial role in the development of the early gut immune system[24]. Th17 cells regulate the secretion of cytokines that promote inflammation in the host, while Treg cells modulate the release of anti-inflammatory cytokines.

**Fig. 1:**
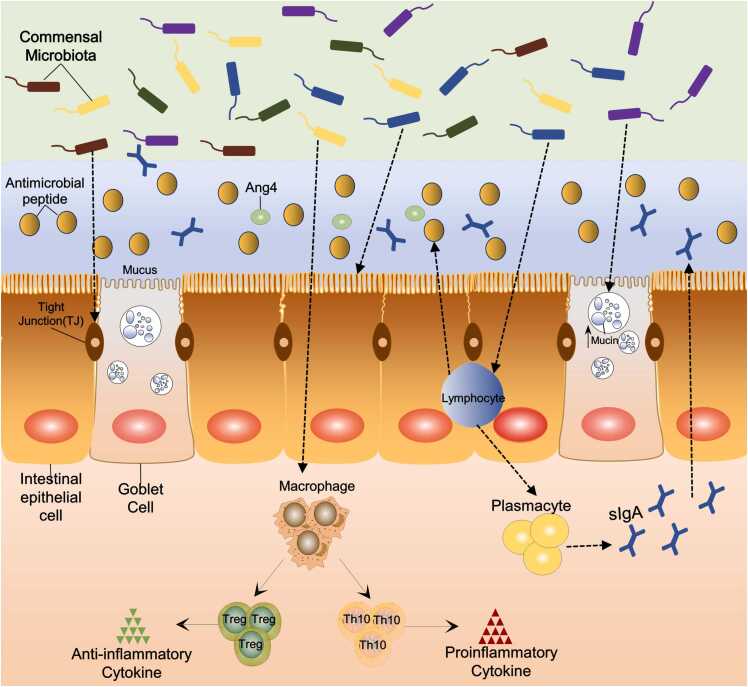
Protective effects of gut microbiota on the intestines. The gut microbiota promotes the development of the intestinal immune system, maintains the balance between Th17 and Treg cells, and induces the release of antimicrobial substances from the immune cells, thereby maintaining immune homeostasis in the intestines. At the same time, gut microbiota regulates the renewal of intestinal epithelial cells, mucus secretion, and the function of tight junctions, thus maintaining the barrier function of the intestines.

Therefore, maintaining the balance between Th17 cells and Treg cells is essential for effective immune function in the gut [25]. When the gut microbiota is dysbiotic, the balance shifts in favor of the Th17 cells, leading to an increased release of inflammation-promoting substances ultimately triggering an inflammatory response in the gut mucosa[26]. Furthermore, antibacterial substances such as secretory globulin A (sIgA)[27] and angiopoietin-4 (Ang4)[28], which are induced by gut microbiota, protect intestinal epithelial cells (IEC) from toxins and pathogenic microorganisms. However, studies have shown that sIgA and Ang4 expression levels are significantly reduced in GF mice. In conclusion, the gut microbiota has a significant influence on the immunological balance in the intestinal mucosa. **(**Fig. 1**)**.

### 2.2 Gut microbiota preserves the gut's barrier integrity

Through two different ways, the gut microbiota may maintain the integrity of the intestinal barrier. First, by controlling the process of epithelial cell renewal and repair, the gut microbiota can protect the intestinal tract barrier's ability to function. Maintaining gut barrier integration depends on ensuring that gut IECs achieve a balance between proliferation and apoptosis. The regeneration of IEC is significantly impacted by a lack of gut microbiota, which eventually causes the intestinal tract's form and function to be disturbed[29]. Compared with SPF mice, GF mice have abnormal gut morphology, abbreviated ileocecal villi, diminished gut saphenous fossa, and a significant reduction in total intestinal surface area[24], [30]. Additionally, the gut microbiota supports the gut barrier by regulating intestinal mucin production[31] **(**Fig. 1**)**. The mucins are necessary to inhibit the attachment or infiltration of luminal bacteria into the intestinal epithelium, a property that is contingent upon the carbohydrate makeup of the mucins. The number of mucin-secreting cup cells in the cecum of GF mice is low compared to that of SPF mice[32]. In contrast, endotoxin treatment by E. coli O55:B5 promoted mucin release in GF mice[33]. In addition, GF mice have different glycosylation characteristics[34]. According to all of the aforementioned research, the gut microbiota plays a critical role in preserving the intestinal barrier's integrity.

## 3 Gut microbiota metabolites protect the intestines

**Fig. 2:**
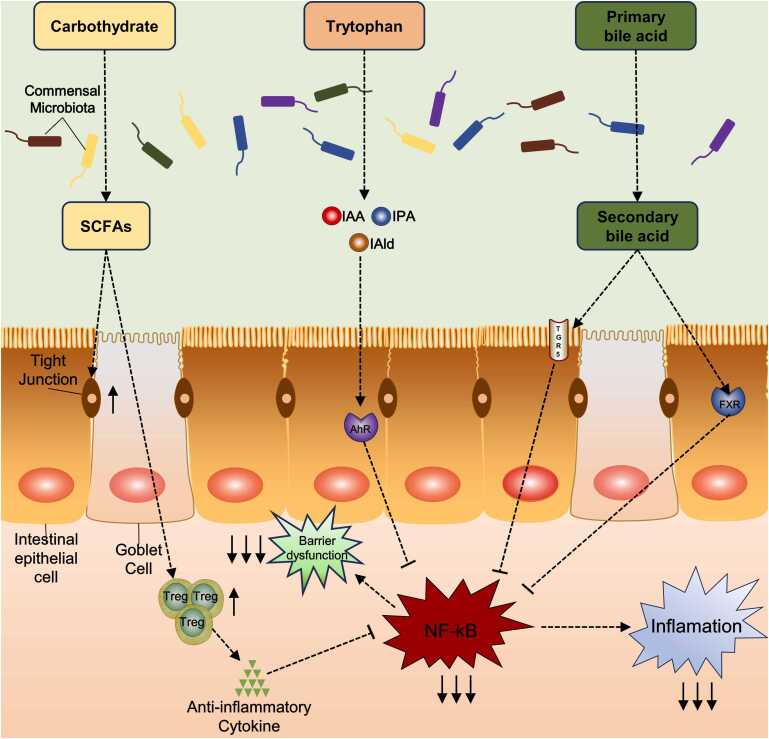
Protective effects of gut microbiota metabolites on the intestines. Gut microbiota metabolites can participate in signal transduction in the intestine, and play a positive role in intestinal barrier protection, immune regulation, nutritional metabolism, etc.

The gut microbiota shows an immense influence on host health, and any perturbation in the host-gut crosstalk may result in a disrupted host health state, while small-molecule metabolites such as SCFAs, Trp, BAs, etc. are messengers for interactions among the host and the gut microbiota[35], [36]. These metabolites can take part in gut signaling and are beneficial for immune modulation, barrier protection, and gut nutrition metabolism **(**Fig. 2**).**

### 3.1 Short-chain fatty acids

SCFAs are among the most abundant bacterial metabolites found in the intestine. They are produced by the gut microbiota in the colon and cecum through the breakdown of indigestible dietary fibers, with primary SCFAs being acetate, propionate, and butyrate[37]. Research has extensively explored the role of SCFAs in modulating the IEC function, influencing intestinal peristalsis, enhancing gut barrier integrity, and improving host metabolism through various mechanisms[38]. One of the key roles of SCFAs, particularly butyrate, is to maintain the integrity of the gut barrier by regulating mucin gene transcription in the goblet cells, which increases the mucus layer[39]. Additionally, butyric acid enhances the function of the intestinal epithelial barrier by modulating the activation of AMP-activated protein kinase (AMPK) leading to the phosphorylation of myosin II-regulated light chain (MLC2), This process promotes the restructuring of tight junctions between epithelial cells[40], [41]. SCFAs also possess strong anti-inflammatory properties, primarily by reducing histone deacetylase (HDAC) activity and upregulating signaling pathways involving the G protein-coupled receptors (GPCRs) [42]. By inhibiting HDACs, SCFAs are believed to promote the growth of Treg cells. Specifically, when HDAC9 is inhibited, the production of FoxP3 is increased, facilitating the differentiation and proliferation of Treg cells which strengthens their protective effect against colitis[43]. Furthermore, butyrate has been shown to limit the synthesis and secretion of proinflammatory cytokines in the colon lamina propria, thereby, reducing the incidence of intestinal inflammation. This effect may be attributed to its role in alleviating the chemotaxis of intestinal neutrophils and down-regulating the expression of proinflammatory cytokines by the specific binding of SCFAs to free fatty acid receptor 2 (FFAR2)[44]. Overall, SCFAs significantly regulate intestinal metabolism, immunity, and barrier function. However, further investigation is needed to clarify their precise mechanism of action

### 3.2 Tryptophan

Tryptophan (Trp) is an essential heterocyclic amino acid, that cannot be synthesized endogenously by the body. It is primarily synthesized by the gut microbiota within the digestive system and serves as a critical component in the host metabolism[45]. Trp is metabolized in the gut through three distinct pathways, each with implications for intestinal health. The first pathway involves the enzymes indoleamine 2,3-dioxygenase (IDO) and tryptophan 2,3-dioxygenase (TDO), which break down Trp through the kynurenine (Kyn) pathway. This process produces several compounds, including kynurenine, quinolinic acid (QA), nicotinic acid, nicotinamide adenine dinucleotide, and kynurenic acid (Kna)[46], [47]. Some of these metabolites play important protective roles in the intestinal tract, with Kna noted for its ability to bind to the G-protein-coupled receptor, GPR35 expressed in the epithelial and immune cells, exerting protective and immunomodulatory effects on the intestinal mucosa[48]. The second pathway is the 5-hydroxytryptamine (5-HT) pathway, in which Trp is taken up by enterochromaffin cells and metabolized by the enzyme Trp hydroxylase 1 (TPH1) into 5-HT[49]. 5-HT influences peristalsis and motility, secretion, vasodilation, and nutrient absorption within the gut. It also interacts with specific 5-HT receptors to convey signals between the gut and neurons, playing. A significant role in regulating intestinal function[50]. The third pathway known as the indole route, involves the direct transformation of Trp into indole and its byproducts, such as indole acid formaldehyde (IAld), indole acetic acid (IAA), indole propionic acid (IPA), indole acetaldehyde (IAAld), and indole acrylic acid， etc.[51]. Indole and its metabolites act as natural ligands for the aryl hydrocarbon receptor (AhR). When activated, the AhR helps reduce the production of pro-inflammatory cytokines, which is beneficial for t intestinal health[52]. The immune response of the gut is significantly influenced by the signaling pathway associated with the AhR. Research indicates that activation of AhR can inhibit the NF-κB signaling pathway by generating a suppressor of cytokine signaling 2 (SOCS2), resulting in anti-inflammatory effects [53]. Moreover, AhR can also contribute to the preservation of the gut epithelial layer of the intestine by stimulating the AhR-Nrf2 pathway or increasing the production of gut-tight junction proteins， including ZO-1 and Occludin[54], [55]. In summary, Trp has a significant impact on barrier protection, immunological homeostasis, and the metabolic regulation of the gut through the above pathways. Nevertheless, given the intricate nature of the intestinal milieu, there is still a need for further studies regarding the interaction between gut microbiota and Trp metabolism.

### 3.3 Bile acid

Hepatocyte-derived BAs, are essential for fat digestion and serve as an important signaling molecule in glucolipid metabolism and immune homeostasis[56]. Over 95 % of synthesized BAs are reabsorbed in the ileum. With only about five percent reaching the colon. In the colon, specific bacterial populations convert primary BAs (PBAs) into secondary BAs (SBAs). SBAs interact with various receptors the G protein-coupled bile acid receptor (TGR5) and the farnesol X receptor (FXR) stimulating their absorption by intestinal epithelial cells (IECs) and playing a vital role in maintaining intestinal homeostasis[57], [58], [59], [60], [61]. Activation of FXR or TGR5 by SBAs is reported to inhibit transcription in the p65 subunit of the NF-κB pathway as well as exert anti-inflammatory effects in the intestine[62]. TGR5 can also modulate gut immunity by influencing macrophage activation[63]. Evidence suggests that TGR5 elicits its distinct response in M1 and M2 macrophages; in M2 macrophages, TGR5 induces IL-10 production, and inhibits TNF-α and INF-γ levels, thereby exerting anti-inflammatory effects[64]. Conversely, in M1 macrophages, TGR5 activation enhances NF-κB expression and pro-inflammatory factor production[65]. Despite the opposing responses in macrophage subsets, TGR5 activation generally has a net anti-inflammatory effect. Moreover, SBA exerts a protective role in preserving the gut barrier by binding to receptors. In one study, it was found that activated FXR could promote regeneration and repair of intestinal crypts by down-regulating elevated prostaglandin E2 levels[66]. Additionally, TGR5 binding enhances IEC regeneration and helps prevent intestinal inflammation[67]. The activation of TGR5 further initiates SRC/YAP signaling pathways that support the renewal and proliferation of intestinal stem cells (ISC) while inhibiting the inflammatory vesicle phosphorylation and ubiquitination of NLRP3 in macrophages as well as exerting protection against gut barrier function[68]. Although extensive research highlights the beneficial effects of SBAs on gut health, it is essential to recognize that not all SBAs confer a protective benefit[69]. For instance, deoxycholic acid has been shown to inhibit colonic epithelial recovery in vivo[70] and is linked to increased neutrophilic infiltration and higher inflammation scores in animal models[71]. The role of SBA in the intestinal environment remains complex and warrants further investigation.

## 4 Modifications and impacts of the gut microbiota and its metabolites in the UC

Increasing evidence indicates that UC onset is closely associated with abnormalities in the gut microbiota and its metabolites. The reduction in both the diversity and abundance of gut microbiota compromises microbial defense mechanisms, facilitating pathogenic bacteria invasion of the intestinal mucosa, breaching the intestinal barrier, and triggering immune dysregulation that promotes the development of colitis[72], [73]. Furthermore, dysbiosis alters levels of key metabolites such as SCFAs, Trp, and bile acids, significantly impacting intestinal immune homeostasis and gut barrier function[74]. Here, we provide an overview of the alterations in gut microbiota and their metabolites in UC and discuss the consequences of the implications for these disturbances on disease pathology.

### 4.1 Gut microbiota in UC: changes and effects

Changes in the quantity and composition of the gut microbiota are indicative of disturbances in the gut environment a. Numerous studies have shown that dysbiosis, or an imbalance in the gut microbiota, both causes and accelerates the onset and progression of UC[89]. Compared to healthy individuals, participants with UC usually show a higher prevalence of harmful microbes and a lower level of beneficial microbes. Specifically, Bacteroidetes and Actinobacteria are more prevalent, while Firmicutes are less prevalent, which is a key characteristic of this condition **(**Table 1**)**. On the one hand, some opportunistic pathogens and pathogenic bacteria, such as Enterobacteriaceae and several pathogenic genera of Proteobacteria, can trigger inflammation, leading to increased production of endotoxin and ultimately accelerating the progression of colitis[90], [91]. On the other hand, a reduction of beneficial bacteria, such as Bacteroidetes, can result in decreased levels of gut antimicrobial peptides, (AMPs) such as REG3G and then DEFB1. This, in turn, weakens the effectiveness of the gut's protective barrier[92]. This damage to the intestinal lining makes the IEC more vulnerable to harmful pathogens. When these pathogens are stimulated by their flagella and lipoproteins, they activate Toll-like receptor 5 (TLR5) on dendritic cells. Colitis develops as a result of this activation, which also causes the synthesis of IL-22 and IL-23[93]. Additionally, an imbalance between Th17 and Treg cells caused by dysbiosis can lead to intestinal mucosal immune diseases, exacerbating colitis[94]. Therefore, gut microbiota dysbiosis disrupts immune homeostasis and the integrity of the gut barrier making it crucial to restore gut microbiota balance as part of UC treatment.

**Table 1:** Changes and effects of gut microbiota in patients with UC.

**Bacterium**	**Changes in UC**	**Effects on UC and microbial metabolites**	**Reference**
Salmonellae	Up	Associated with immune response and hypersensitivity status in UC	[75]
Escherichia coli	Up	Promotes the release of inflammatory cytokines TNF-α, IL−6 and IL−23	[9], [76], [77]
Shigella	Up	Causes severe human intestinal syndrome through its type III secretion system (T3SS)	[78]
C. difficile	Up	Produces toxic cytokines that damage the intestinal epithelial barrier and induce a strong inflammatory response	[79], [80]
Sulfate-Reducing bacteria	Up	Promotes increased intestinal permeability	[81]
Bifidobacterium	Down	Produces SCFAs to protect the intestinal barrier	[82], [83]
Lactobacillus	Down	Generation of AhR ligands to protect the intestinal barrier	[80], [84]
Akkermansia	Down	Produces SCFAs to protect the intestinal barrier	[85], [86]
Ruminococcus	Down	Alleviation of colonic inflammation by activation of NLRP3 and reduction of inflammatory cytokines	[87]
Saccharomyces boulardii	Down	Produces SCFAs to protect the intestinal barrier	[88]

### 4.2 Gut microbiota metabolites in UC: changes and effects

#### 4.2.1 SCFAs and UC

Overall, there is a reduced presence of SCFA-producing bacteria in individuals with UC. Acetate is mainly metabolized by Akermannia and Bacteroides[38]; propionate is primarily metabolized through Bacteroidetes as well as Firmicutes[95]; and butyrate is generated by four main producers, which are Eubacteriaceae, Bacteroidetes, Lactobacilli, and Vibrionidae[96], [97]. The decrease in SCFA producers has also altered, the gut levels of acetate, butyrate, and propionate (Table 2). Fecal propionate levels were lower in dysbiosis patients when compared to non-dysbiosis subjects, while the decrease in butyrate coincided well with the reduced abundance of butyrate-producing bacteria Thus, intestinal acetate, butyrate, and propionate levels were also changed[9]. Patients with UC, at various phases of the disease, show decreases in both butyrate and acetate in their feces. Notably, in patients with active UC, the decrease in butyrate was accompanied by a decrease in the number of butyrate-producing bacteria. However, these butyrate producers start to recover as evidenced by increased butyrate levels in samples taken during remission [98]. In addition, evidence from 16sRNA sequencing suggests that patients with UC have significantly increased numbers of Salmonella and G provotella in their feces, leading to a decrease in butyrate level in the intestine and exacerbating the gut barrier damage in the colon[99], [100]. This reduction in butyrate may contribute to the inhibition of intestinal epithelial tight junction protein secretion[101**]** (Fig. 2). In conclusion, SCFA levels are decreased in patients with UC, and exogenous supplementation of SCFAs may potentially offer a treatment avenue in the future. However, the results of SCFA supplementation in a mouse model of colitis showed that the symptoms of colitis were not improved. This lack of improvement may be related to the way SCFA is supplemented in the experiments, the concentration of butyrate, or the specific disease model employed[102**]**. Therefore, further exploration is needed to clarify the mechanisms and specific role of SCFAs in UC.

**Table 2:** Main clinical trials of FMT in the treatment of UC.

**Year of publication**	**Classification of UC**	**Sample size**	**Administration mode of FMT**	**Single dose**	**Frequency**	**Treatment outcomes**
2015[129**]**	Mild to moderate	50	Placement of nasoduodenal tube by Cortrak method or endoscopy	50 ml	At 0 and 3 weeks	In the FMT group, 30 % of patients had an endoscopic response at week 2
2015[112**]**	Mild to severe	75	Enema	50 ml	Once a week for 6 weeks	Symptom resolution at week 2 occurred in 24 % of the FMT group
2017[130**]**	Mild to moderate	85	Placement of nasoduodenal tube by endoscopy	150 ml	Twice in 3 weeks for 12 weeks	Symptom resolution at week 2 occurred in 27 % of the FMT group
2017[131**]**	Mild to moderate	41	Colonoscopic	500 ml	single-pass	No clinical remission in the FMT group at 8 weeks
2017[132**]**	Mild to severe	36	Colonoscopic	350–500 ml	single-pass	After 4 weeks of FMT treatment, 53.0 % of the patients had clinical remission
2019[133**]**	Mild to moderate	73	Enema and colonoscopic	200 ml	Twice in 7 days	After 8 weeks of FMT treatment, 32.0 % of the patients had clinical remission
2019[134**]**	Active UC	81	Enema		5 d/wk for 8 weeks	Patients in remission after receiving FMT were enriched in Eubacterium hallii and Roseburia inulivorans and had increased levels of SCFAs biosynthesis and secondary BAs.
2019[135**]**	Clinical remission	61	Colonoscopic	200 ml	Every 8 weeks for 48 weeks	Maintenance FMT in patients who are in clinical remission may help sustain clinical, endoscopic and histological remission in patients with UC.
2020[136**]**	Moderate to severe	16	Colonoscopic	500 ml	Three times at 2–3 month intervals	Serum concentrations of IL−1Ra, IL−6, IP−10, ENA−78, MEC, VCAM−1, and G-CSF decreased significantly after FMT.
2022[137**]**	Mild to moderate	66	Colonoscopic	200–250 ml	7 d/wk for 8 weeks	Multidonor FMT with anti-inflammatory diet effectively induced deep remission in mild-moderate UC which was sustained with anti-inflammatory diet over 1 year.
2022[138**]**	Mild to moderate	122	Enema and colonoscopic	150 ml	3 days	FMT therapy was as effective as glucocorticoids to induce remission in active mild to moderate UC, accompanied by fewer adverse events.
2023[139**]**	Mild to severe	31	Oral	30 capsules	Once every two days for six days	Capsule FMT improved microbial fungi diversity and change of the fungus
2023[140**]**	Clinical remission	48	Colonoscopic	250 ml	single-pass	Single-dose FMT cannot be used to maintain UC remission

#### 4.2.2 Trp and UC

According to a growing body of recent research, the development of UC is intrinsically connected to abnormalities in Trp metabolism[103], [104]. One clinical study found that Trp levels were reduced in patients, with its concentrations inversely correlated with disease severity[105**]**. This may be related to Trp participation in multiple intestinal metabolic pathways, where its deficiency directly leads to metabolic imbalances, significantly affecting intestinal homeostasis and the severity of UC. Studies have shown that administration of Trp and its partial metabolites alleviated colitis symptoms in UC models of mice, whereas removal of Trp from the diet increased disease susceptibility[105**]**. Furthermore, an excessive lack of Trp will result in an increase in Kyn synthesis during Trp metabolism, preventing effector T cells and natural killer cells from proliferating and functioning. The ratio of Kyn to Trp correlates positively with disease severity in UC[106**]**. Trp and its metabolites might therefore be novel targets for UC therapy in the future.

#### 4.2.3 BAs and UC

In general, there is a balance between PBA and SBA, but gut microbiota dysbiosis disrupts it, and studies have shown that fecal PBA concentrations in UC patients are significantly higher than those in normal people, whereas SBA concentrations are significantly lower than those in normal people[107**]**. As a high-affinity ligand for FXR as well as TGR5, this alteration of SBA inhibits TGR5 as well as FXR activation, which ultimately leads to the decrease of anti-inflammatory effects[108**]**. In contrast, when SBA is restored it relieves UC symptoms, the mechanism of which may be related to the reduction of neutrophil infiltration in colonic tissues[109**]**. Additionally, it has been shown that diarrhea in UC patients may be related to BA malabsorption, and the higher the fecal BA concentration, the larger the area of colonic damage[110**]**. Therefore, regulating the balance of BA metabolism will be a new direction for treating UC.

## 5 Treatment of UC based on regulation of gut microbiota and its metabolites

**Fig. 3:**
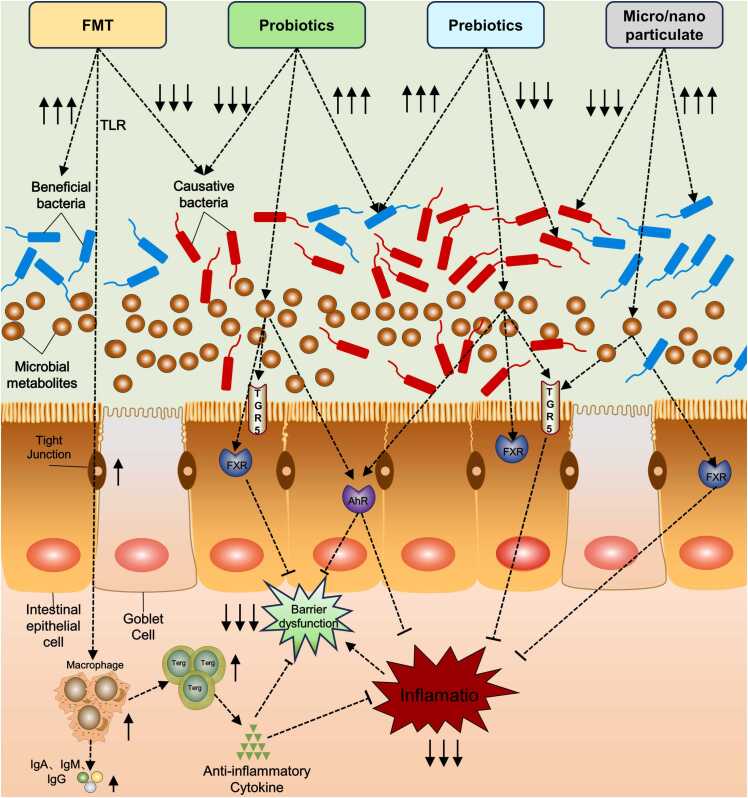
Treatment of UC by modulation of gut microbiota and its metabolites. FMT, probiotics, prebiotics and Micro/nanoparticles restore the balance of gut microbiota by inhibiting harmful bacteria and promoting beneficial bacteria. In addition, FMT promotes the production of immunoglobulins and anti-inflammatory cytokines through the TLR pathway, alleviates intestinal inflammation and protects the integrity of the intestinal barrier. Instead, probiotics can function as anti-inflammatories and protect the gut barrier by modulating gut microbiota metabolites. While probiotics, prebiotics and Micro/nanoparticles can also be involved in signaling in the gut by modulating gut microbiota metabolites, inhibiting inflammatory responses and protecting the intestinal barrier.

The physiological functions of the intestine may be restored by returning the intestinal microbiota and its metabolites to their initial state, even though further research is needed to determine the roles that different gut microbiota and their metabolites play in the host gut. Indeed, in recent years, research has focused on UC treatment approaches that regulate the gut microbiota and its metabolites[111**]**. Fecal microbiota transplantation (FMT) has been demonstrated to be used to correct dysbacteriosis and has shown satisfactory effects in alleviating UC[112**]** **(**Fig. 3**)**. Similarly, the gut microbiota and its metabolites are regulated by prebiotics and probiotics, which may be used as UC treatments[113**]**. Moreover, the emergence of Micro/nanoparticles in recent years has also shown potential for the treatment of UC and has been noticed for its outstanding colonic targeting[114**]**. To provide a theoretical foundation for UC therapy, we will present each of these four approaches in the following sections. These approaches include altering gut microbiota or its metabolites.

### 5.1 Fecal microbiota transplantation

To restore intestinal flora balance and make the patient's intestinal flora more like that of the donor over an extended period, FMT involves transferring fecal bacteria from a healthy donor into the patient's distal gastrointestinal canal. By stimulating immune cells to release IgA, IgG, and IgM via the TLR pathway, FMT may help to preserve intestinal homeostasis by triggering the intestinal humoral immune response **(**Fig. 3**)**. Additionally, by lowering intestinal pH and reducing bacterial and hydroperoxide adhesion, it can competitively restrict the adherence and transmission of harmful microbes[115**]**. While FMT is increasingly being utilized to treat recurring C. difficile infections[116**]**, the first study using FMT in treating UC appeared as early as 1989[117**]**. This investigator later noted in a review that patients initially treated had no signs of colonic inflammation when examined endoscopically and histologically for more than 20 years following the treatment [118**]**. An increasing number of studies have been conducted in recent years to confirm the therapeutic viability of FMT for the treatment of UC[119], [120] **(**Table 2**)**. Costello et al.[121**]** reported a case in which the patient's UC continued to recur after treatment with mesalazine, azathioprine, and infliximab. Hence the patient was given FMT, and symptomatic relief was observed after 8 weeks, with endoscopic visualization of UC inflammation subsiding. In addition, continued follow-up with this patient showed that even after 12 months, the patient retained clinical and endoscopic remission of UC, demonstrating an excellent therapeutic outcome. At the same time, in a prospective, uncontrolled study of 20 people, FMT therapy changed the gut microbiota abundance of patients, significantly alleviating clinical symptoms, and partially reducing colonic mucosal lesions[122**]**^.^ Furthermore, Dutta[123**]** et al. found an increase in the proportion of Lachnospiraceae, the butyrate-producing bacteria, after FMT treatment of patients. These findings confirm that FMT's may act by modulating the gut microbiota, while further suggesting that the promotion of Lachnospiraceae may be key to the success of FMT. Lachnospiraceae may be a crucial bacteria for the success of FMT, according to this study, which also supports the potential mode of action of FMT. This finding may lead to more uniform and focused FMT. FMT has demonstrated encouraging results for remission in UC patients; however, its safety and maintenance impact have not been thoroughly assessed[124**]**. Side effects such as bacteremia[125**]**, alterations in the immune function of the intestinal mucosa[126**]**, altered intestinal ecosystems[127**]**, and metabolic differences in the organism[128**]** have been reported to be possible as a result of FMT. This suggests that further study is required to show that FMT is safe and effective in treating UC and to better understand how FMT works as a treatment.

### 5.2 Probiotics

Live bacteria that colonize the host and improve its health are known as probiotics[141**]**. Common genera include Lactobacillus, Bifidobacterium, and Enterococcus, among others. Recent studies have shown that probiotics, as an intestinal bacterium regulator, can cure UC by balancing the microbiota **(**Table 3**)**. Akkermansia muciniphila is a novel probiotic that can increase the abundance of Firmicutes, restore the gut microbiota structure in DSS-induced colitis mice, reduce inflammation levels through microbial-host interaction, and protect the mucosal barrier[142**]**. Probiotics can also protect the UC by increasing the amount of metabolites in the flora. According to research, Bifidobacterium can reduce inflammation in colitis-affected animals by increasing the synthesis of SCFAs[143**]**, which may help to maintain gut homeostasis and reduce inflammation by producing the butyrate, a metabolite produced by the gut microbiota[144**]**. Furthermore, when probiotics are used in conjunction with traditional drugs that reduce inflammation, the effects appear to be better than when probiotics are used alone to treat UC. Chapman et al.[145**]** demonstrated that in the treatment of UC patients, compared with the non-combination group, the combination of probiotics mixture and 5-ASA can shorten the recovery time of UC, weaken the disease activity, and show better therapeutic effects under endoscopy. Although the successful outcome of probiotics in treating UC is promising, there remain certain safety concerns. Nissle 1917 Escherichia coli reduces UC symptoms brought on by inflammatory agents including TNF-α, IL-6, and IL-1β[146**]**. According to recent research, there is a pks pathogenic island in the genome of Escherichia coli Nissle 1917 that may be connected to the development and spread of colorectal cancer[147**]**. Therefore, using probiotics for the treatment of UC requires a more in-depth exploration of their safety, in addition to more supportive data on their therapeutic efficacy.

**Table 3:** Main clinical trials of Probiotics in the treatment of UC.

**Year of publication**	**Strain**	**Classification of UC**	**Sample size**	**Treatment outcomes**
2013[148**]**	Bifidobacterium infantis 35624	Mild to moderate	22	Reduction in the levels CRP and TNF-α in both gastrointestinal and non-gastrointestinal inflammatory disorders, but did not particularly affect UC disease.
2014[149**]**	Escherichia coli Nissle 1917	Active UC	100	The use of E. coli Nissle as an add-on to conventional therapy for active ulcerative colitis is not beneficial.
2015[150**]**	Streptococcus faecalis T−110, Clostridium butyricum TO-A and Bacillus mesentericus TO-A	Clinical remission	60	Probiotics may be effective for maintaining clinical remission in patients with quiescent UC, especially those who belong to cluster I on fecal bacterial analysis.
2015[151**]**	Bifidobacterium longum 536	Mild to moderate	56	Supplementation with BB536 was well tolerated and reduced UCDAI scores, EI and Mayo subscores after 8 weeks in Japanese patients with mild to moderately active UC.
2016[152**]**	Lactobacillus salivarius, Lactobacillus acidophilus and Bifidobacterium bifidus strain BGN4	Moderate to severe	60	Reduction in recovery time, weaker activity of the disease, better endoscopic picture.
2018[153**]**	Bifidobacterium breve Fermented Milk	Quiescent UC	195	No beneficial effect
2019[154**]**	Lactobacillus rhamnosus NCIMB 30174, Lactobacillus plantarum NCIMB 30173, Lactobacillus acidophilus NCIMB 30175 and Enterococcus faecium NCIMB 30176	Quiescent UC	81	This multi-strain probiotic is associated with decreased intestinal inflammation in patients with UC, but not in CD and is well tolerated.
2022[155**]**	Escherichia coli Nissle 1917	Mild to moderate	133	Patients with mild to moderate UC have endoscopic remission with reduced IBDQ scores.
2023[156**]**	Lactobacillus paracasei (A234),Lactobacillus gasseri (A237),Lactobacillus rhamnosus (A193),Lactobacillus acidophilus (A118),Lactobacillus plantarum (A138),Lactobacillus casei (A179),Lactobacillus reuteri (A113),Lactococcus lactis (A328),Bifidobacterium animalis subsp.lactis (A026),Bifidobacterium breve (A055),ifidobacterium long subsp. Longum (A027),Bifidobacterium bifidum(A058),Bifidobacterium long subsp. infantis (A041)	Mild to moderate	24	The use of probiotic therapy containing Lactobacillus and Bifidobacterium species had significantly improved the quality of life among UC patients

### 5.3 Prebiotics

Prebiotics are a class of organic compounds that are specifically broken down and utilized by the beneficial microbiota of the human gut[157**]**. This group mainly consists of functional oligosaccharides, such as fructooligosaccharides, galactooligosaccharides, and inulin. Prebiotics can stimulate the proliferation of endogenous intestinal microflora, thereby enhancing intestinal function[158], [159]. Exogenous probiotic supplementation has been shown to suppress the growth of pathogenic bacteria in the intestinal tract and encourage the spread of beneficial bacteria, which helps patients' intestinal microbiotas return to equilibrium in several UC models. Inulin, for example, can increase the number of lactic acid bacteria in the colon and lower the intestinal pH. This environment stops the growth of bacteria from growing and alleviates symptoms of colitis[160**]**. The application of inulin and oligofructose in healthy populations has been shown to increase the abundance of lactic acid bacteria in the intestine[161**]**. Furthermore, prebiotics can aid in the treatment of UC by altering the metabolites of the gut microbiota, such as resistant starch (RS). RS is an anti-enzymatic starch that the gut microbiota can utilize to boost the number of bacteria that produce SCFAs in the gut[162**]**. Additionally, sprouted glutamine and hemicellulose-rich barley foods are metabolized to butyrate by Eubacterium and Bifidobacterium[163**]**, which in turn plays a role in repairing the intestinal barrier and alleviating colonic inflammation[164], [165]. While research on prebiotics that can alleviate colonic inflammation in UC is expanding, it may be unrealistic to expect that prebiotics alone can cure UC. However, prebiotics offer several advantages -they are easy to consume, cost-effective, and biocompatible- making them a therapeutic adjunct suitable for long-term use.

### 5.4 Micro/nanoparticulate

Micro and nanoparticles mainly include microparticles (MPs) and nanoparticles (NPs). Due to their unique characteristics- such as wide size range, adjustable shape, and ease of modification, it is gaining more attention in the medical field, particularly for disease diagnosis and treatment[166], [167], [168], [169], [170]. Recent studies have revealed that some micro- and nanoparticles can play a protective role against UC by modulating gut microbiota or metabolites **(**Table 4**)**.

**Table 4:** Micro/nanoparticles for the treatment of UC based on modulation of gut microbiota.

**Year of publication**	**Micro/nanoparticulate**	**Mechanism**	**Treatment outcomes**
2014[183**]**	Redox nanoparticle	Scavenging of ROS, suppression of inflammatory responses, and inhibition of commensal bacterial translocation/infection.	It protects the intestinal mucosa and relieves intestinal inflammation.
2017[184**]**	Theracurmin	Increase butyrate bacteria abundance and butyric acid salt content in feces.	Treatment with nanoparticle curcumin significantly attenuated body weight loss, disease activity index, histological colitis score and significantly improved mucosal permeability.
2018[185**]**	AuNP	Decrease the α-diversity, the Firmicutes/Bacteroidetes ratio, certain short-chain fatty acid-producing bacteria and Lactobacillus.	It has a more pronounced anti-colitis effect.
2019[186**]**	CS-CUR-NPs	Regulating gut microbiota and targeting CD44 to enhance the uptake ability of macrophages.	Alleviate the inflammation of the colon.
2019[187**]**	TiO2 NPs	Interfering with the balance of the immune system and the dynamics of the gut microbiota.	TiO2 NPs aggravated DSS-induced chronic colitis and immune response in vivo, and reduced the population of CD4 +T cells, regulatory T cells, and macrophages in mesenteric lymph nodes.
2020[188**]**	Mesoporous silica capped with the cucurbituril complex	Restores Trp metabolism of the microbiota and induces activation of AHR, thereby enhancing anti-inflammatory effects.	Promoted the colon epithelial barrier integrity, improve the level of the AHR agonist, and regulate the production of inflammatory cytokines.
2021[189**]**	MDC@MCNs	It can effectively inhibit inflammation and oxidative stress, maintain close connection of colon and regulate gut microbiota.	The injury of colonic epithelial cells was alleviated, and the colonic inflammation was subsided.
2021[190**]**	Berberine-Loaded Carboxylmethyl Chitosan Nanoparticles	Regulation of IL−6 expression levels and remodeling of gut microbiota.	Significantly improve the colon inflammation and damage of the colon.
2021[191**]**	GEN-NP2	Inhibit the secretion of IL−1β and TNF-α, and regulate the gut microbiota.	Alleviates colonic inflammation.
2021[192**]**	HA-CG-CO 2 @NPs	It alleviates CD98-mediated epithelial barrier dysfunction, down-regulates proinflammatory cytokines, and improves microbial dysbiosis.	The intestinal barrier was restored and inflammation was reduced.
2022[193**]**	Rh2NPs	Improve the species uniformity and abundance of gut microbiota, restore the species diversity of gut microbiota, and effectively regulate the gut microbiota of mice.	The treatment ameliorated DSS -induced weight loss, colon length, disease activity index (DAI) score and myeloperoxidase (MPO) activity in mice.
2022[194**]**	Turmeric-derived nanovesicles	Regulate gut microbiota and promote the transformation of M1 phenotype into M2 macrophages.	To restore the damaged intestinal epithelial barrier, colitis resistance effect into full play.
2022[195**]**	HPN-NE-EcN	Restoring the abundance and diversity of gut microbiota.	It plays a role in the prevention and treatment of DSSS-induced colitis.
2023[196**]**	PELN	Enhanced L. reuteri growth and elevated levels of indole derivatives, which may activate the AhR in conventional CD4 + T cells. This activation down-regulates Zbtb7b expression, leading to the reprogramming of conventional CD4 + T cells into double-positive CD4 +CD8 +T cells.	Alleviates DSS-induced colon inflammation in mice.
2023[197**]**	Rh 2/LA-UASP NPs	Restore the normal levels of gut microbiota and SCFAs, inhibit the release of proinflammatory factors.	Significantly improved the integrity of the intestinal mucosa and increased colon length.
2023[198**]**	BBRNPs	Inhibition of NF-κB/STAT−3 pathway activation and increased expression of tight junction proteins in vivo	The symptoms of colitis were alleviated in mice.

NPs can attach directly to pathogenic bacteria in the intestines, regulating the gut bacteria. Metal NPs are nanomaterials created by encasing metal cores in metal oxides or inorganic materials. Their broad-spectrum antibacterial properties make them effective for treating intestinal pathogen infections[171**]**. For instance, silver nanoparticles (AgNPs), synthesized using plants and silver salts, have been shown to have antimicrobial effects against E. coli and Micrococcus luteus, two harmful intestinal bacteria. This efficacy is largely attributed to the fact that many harmful bacteria possess Ag-binding proteins, allowing AgNPs to impair enzymatic function by binding to the residue His185 [172**]**. Similarly, copper nanoparticles (CuNPs) and zinc nanoparticles (ZnNPs) exhibit antimicrobial activities, with ZnNPs capable of bactericidal action by interacting directly with the phospholipid bilayer of the bacterial cell membrane, leading to their loss of integrity[173], [174]. Notably, the mechanism by which metal NPs exert their antimicrobial effect is vastly different from those of antibiotics, and concerns regarding drug resistance in gut microbiota are minimized. Moreover, some studies have indicated that combining antibiotics with metal NPs can reduce the required antibiotic dosage and decrease bacterial resistance[175**]**.

MPs have also attracted attention due to their advantages in providing protection and facilitating targeted, controlled release. MPs can act as carriers to shield antibodies or probiotics that are otherwise easily degraded in the gastric environment, ensuring their safe delivery to the colon. Once in the colon, MPs can then participate in modulating the gut microbiota and metabolites[176**]**. For example, Li et al.[177**]** encapsulated monoclonal antibodies in hydrogel microcapsules, which protected the antibodies from being destroyed by the harsh gastrointestinal environment. In a UC animal model, oral treatment with these antibody-loaded hydrogel microcapsules significantly reduced colitis symptoms and increased populations of beneficial bacteria, such as Clostridium, Enterobacter, and Spirochetes lardii. In addition, Wang et al.[178**]** developed a nitric oxide (NO) responsive polygam-glutamate hydrogel microcapsule loaded with probiotics, which enabled the probiotics to be transported to the intestine smoothly. At the same time, the microcapsule could respond to NO stimulation, rapidly release probiotics, regulate the balance of gut microbiota, protect the intestinal mechanical barrier, and have obvious therapeutic effects on colitis. Additionally, microcapsules made from prebiotics or Biostime as raw materials not only target drugs for anti-inflammatory effects but also help regulate gut microbiota and its metabolites providing relief from colon inflammation. Kaur et al.[179**]** developed a microcapsule loaded with mesalazine, using a shell made of guar gum and xanthan gum as raw materials. These polysaccharides could be degraded by gut microbiota and serve as prebiotics, to regulate the flora and its metabolites. Recently, Yang et al.[180**]** proposed a novel co-delivery microcapsule of prebiotics and epigenetic elements, encapsulating indole-3-propionic acid, a known epigenetic element, with sodium alginate, resistant starch, and chitosan.

Recent developments have even led to the encapsulation of nanoparticles in microspheres. This design offers additional benefits, such as the ability for NPs to accumulate in diseased tissues and be protected and released in a targeted manner by MPs. This has a lot of potential for the future development of oral medication delivery systems that specifically target the colon[181], [182]. To summarize, micro and nanoparticles hold significant potential in the treatment of UC by influencing the composition and metabolites of the gut flora. To enhance the accuracy of UC treatment, it is necessary to develop more micro/nanoparticles that specifically target the main microorganisms and their byproducts at the site of inflammation.

## 6 Conclusions

The above therapies may be the key to ending the impasse in UC treatment since they have a significant impact on the host's gut microbiota and its metabolites. Because of the diversity of the gut microenvironment, it is still very difficult to determine the effects of the entire gut microbiota and its metabolites. To solve this problem, we need a larger cohort of UC patient samples as well as channels for detecting gut microbiota and its metabolites. Additionally, it is essential to create a comprehensive database that combines macrogenomics and bioinformatics technologies. This will help identify the specific molecular mechanisms through which various gut microbiota and their metabolites operate, by thoroughly analyzing and validating the data. A deeper understanding of how the microbiota and their by-products influence human health– at molecular, cellular, and tissue levels– along with how the host interacts with the gut microbiota, will allow us to identify patients at high risk before they exhibit clinical symptoms. This knowledge also paves the way for developing tailored treatment plans for patients with UC who share a similar profile of the disease. Furthermore, there are still challenges regarding the administration, safety, and efficacy of modifying gut microbiota and its metabolites to treat UC. More studies should be conducted in vitro, and in vivo as well as translational studies are necessary to determine its indications, safe dosages, and potential adverse reactions. These studies will lay the groundwork for future clinical applications. In conclusion, targeted modulation of the gut microbiota and its metabolites presents a promising treatment option for UC, but further research is required.

## Funding

This research was funded by the Shaanxi Province Key Research and Development Program (2023-YBSF-072 and 2024JC-YBMS-664), the Innovation Team of Xi'an Medical University (2021TD15), Xi'an Science and Technology Plan Project (24YXYJ0143), Special Service Projects of the Education Department of Shaanxi Province (24JC079); Supporting Project of the First Affiliated Hospital of Xi'an Medical University (XYFYPT-2023-04).

## Author Statement

We declare that this manuscript is original, has not been published before and is not currently being considered for publication elsewhere.

We confirm that the manuscript has been read and approved by all named authors and that there are no other persons who satisfied the criteria for authorship but are not listed. We further confirm that the order of authors listed in the manuscript has been approved by all of us.

We understand that the Corresponding Authors are Manli Cui and Mingxin Zhang, who are the sole contacts for the Editorial process. They are responsible for communicating with the other authors about progress, submissions of revisions and final approval of proofs.

## CRediT authorship contribution statement

**Li Huanyu:** Writing – original draft, Validation, Software, Methodology, Investigation, Formal analysis. **Li Yifan:** Methodology, Formal analysis, Conceptualization. **Pan Meng:** Writing – review & editing, Investigation. **Zhang Mingxin:** Writing – original draft, Supervision, Project administration, Funding acquisition, Conceptualization. **Cui Manli:** Writing – review & editing, Supervision, Project administration.

## Declaration of Competing Interest

The authors declare that they have no known competing financial interests or personal relationships that could have appeared to influence the work reported in this paper.
